# Agreement between temperature measurements during cooling and warming through the esophagus

**DOI:** 10.1186/cc12252

**Published:** 2013-03-19

**Authors:** EB Kulstad, DM Courtney, P Shanley, A Metzger, T Matsuura, J Rees, K Lurie, S McKnite

**Affiliations:** 1 Advocate Christ Medical Center, Oak Lawn, IL, USA; 2 Northwestern University, Chicago, IL, USA; 3 University of Minnesota Medical Center, Minneapolis, MN, USA; 4 Advanced Circulatory Systems, Minneapolis, MN, USA; 5 Minneapolis Medical Research Foundation, Minneapolis, MN, USA

## Introduction

Measurement of temperature during treatment with therapeutic hypothermia can yield variable values depending on where temperature is measured. Measurements of temperature can be made rectally, intravascularly, vaginally, or from the bladder, but agreement between these sites is often uncertain. We measured temperature at multiple sites during experimental induction and reversal of hypothermia in swine with a novel esophageal cooling device, hypothesizing that agreement between sites would fall within standard acceptance criteria (average difference ± 1.96 standard deviation of the difference) in Bland-Altman analyses.

## Methods

Five female Yorkshire swine (mean weight 65 kg) were anesthetized and cooled for 24 hours, then gradually rewarmed, with a novel esophageal heat transfer device powered by an external chiller (Gaymar MediTherm III). Swine temperature was measured intravascularly, rectally, and either vaginally or in the bladder. Comparisons between temperature readings were then made via Bland-Altman plots.

## Results

Agreement between the different sources of temperature measurement was generally high, with less than 10% outliers beyond ±1.96 SD. The best agreement was seen between intravascular temperature measurement and bladder temperature measurement (SD = 0.14°C), with vaginal measurements showing less agreement (SD = 0.23°C), and rectal measurements showing the least (SD = 0.31°C) with intravascular.

## Conclusion

Bladder temperature measurements demonstrated the best agreement with intravascular temperature measurements during cooling with an esophageal heat transfer device; however, reasonable agreement was demonstrated with rectal and vaginal temperature measurements, suggesting that these sites are also acceptable for use.
